# The psychosocial difficulties in brain disorders that explain short term changes in health outcomes

**DOI:** 10.1186/1471-244X-13-78

**Published:** 2013-03-11

**Authors:** Alarcos Cieza, Cristina Bostan, Jose Luis Ayuso-Mateos, Cornelia Oberhauser, Jerome Bickenbach, Alberto Raggi, Matilde Leonardi, Eduard Vieta, Somnath Chatterji

**Affiliations:** 1 Faculty of Social and Human Sciences, School of Psychology (Building 44), University of Southampton, Highfield Campus, Southampton, SO17 1BJ, UK; 2 Department of Medical Informatics, Biometry and Epidemiology – IBE, Chair for Public Health and Health Services Research, Research Unit for Biopsychosocial Health, Ludwig-Maximilians-University (LMU), Munich, Germany; 3 Swiss Paraplegic Research, Nottwil, Switzerland; 4 Department of Psychiatry, Hospital Universitario de la Princesa, Universidad Autonoma de Madrid, CIBERSAM, Madrid, Spain; 5 Neurological Institute C. Besta IRRCS Foundation – Neurology, Public Health and Disability Unit, Milan, Italy; 6 Bipolar Disorders Program, Institute of Neuroscience, Hospital Clinic, University of Barcelona, IDIBAPS, CIBERSAM, Barcelona, Spain; 7 Multi-Country Studies, Department of Measurement and Health Information Systems, World Health Organization, Geneva, Switzerland

**Keywords:** Psychosocial, Psychiatric disorders, Neurological disorders, Disability

## Abstract

**Background:**

This study identifies a set of psychosocial difficulties that are associated with short term changes in health outcomes across a heterogeneous set of brain disorders, neurological and psychiatric.

**Methods:**

Longitudinal observational study over approximately 12 weeks with three time points of assessment and 741 patients with bipolar disorders, depression, migraine, multiple sclerosis, parkinson’s disease, stroke and traumatic brain injury. The data on disability was collected with the checklist of the International Classification of Functioning, Disability and Health. The selected health outcomes were the Short Form 36 and the World Health Organization Disability Assessment Schedule. Multilevel models for change were applied controlling for age, gender and disease severity.

**Results:**

The psychosocial difficulties that explain the variability and change over time of the selected health outcomes were energy and drive, sleep, and emotional functions, and a broad range of activities and participation domains, such as solving problems, conversation, areas of mobility and self-care, relationships, community life and recreation and leisure.

**Conclusions:**

Our findings are of interest to researchers and clinicians for interventions and health systems planning as they show that in addition to difficulties that are diagnostic criteria of these disorders, there are other difficulties that explain small changes in health outcomes over short periods of time.

## Background

The overall prevalence of brain disorders – by which we mean neurological and psychiatric conditions – is very high in Europe [1]. Twenty-seven percent of the European population 18–65 years old are or have been affected by at least one brain disorder in the past 12 months. In population terms, it is estimated that between 78.5 and 87.1 million persons are affected, and about one third of persons with brain disorders have more than one [2].

Cost-of-illness studies consistently show that the economic and social costs of brain disorders are enormous and are, for example, considerably larger than costs of diabetes or cancer [3]. Although it is well known that these costs are high, nonetheless there is also evidence that the overall, personal, social and economic costs of brain disorders have been underestimated for decades because of the lack of valid and relevant information [1,3,4]. Cost-of-illness studies usually do not include the full range of psychosocial difficulties that actually shape the experience of persons with these disorders, affect their quality of life and determine the scope and frequency of clinical interventions. Getting this information is therefore vital and this study will focus on identifying psychosocial difficulties that are associated with a heterogeneous range of brain disorders.

The Global Burden of Disease (GBD) study [5] also highlighted the underappreciated significance of the psychosocial burden of living with brain disorders and made clear that difficulties in participating in personal, family and social life greatly contribute to the burden of brain disorders. The WHO’s 2006 report on neurological disorders [6] notes that not only the burden of these disorders for the person, but also the impact on families and communities have to be taken into account. Moreover, the burden of living with neurological disorders is heightened by widespread stigma and discrimination created by the attitudes of those around the individual, both friends and strangers. These conclusions are supported by the country-by-country data collated in WHO’s 2005 Mental Health Atlas [7]. This states that the single most important research need for intervention planning and management is epidemiological data that is relevant, not merely to the prevalence of brain disorders, but to the prevalence of psychosocial problems that people with these disorders will experience over their lives. This was also supported by WHO’s 2004 Atlas for Neurological Disorders [8].

There are a few European studies that include descriptions and assessments of psychosocial difficulties associated with brain disorders [1]. Some of these studies have used the WHO’s *International Classification of Functioning, Disability and Health* (ICF) [9] as the framework for describing psychosocial difficulties of brain disorders, which in the ICF are called “disabilities” [10-14]. Since the ICF is an etiologically neutral classification, it has the great advantage that it can be used for describing the psychosocial difficulties of any brain disorder. Based on the need to carry out studies that go beyond the common practice of focusing on a single brain disorder, or combinations of two of them [1,15], our understanding of psychosocial difficulties associated with brain disorders can take advantage of the ICF to structure a systematic description of these difficulties. In addition, the ICF also includes environmental factors, understood as determinants that can have a potential positive or negative influence on health outcomes. In other words, they can be facilitators or barriers. The use of the ICF for data collection is thus further justified as it allows for the identification of environmental determinants.

There is also a need for studies that address psychosocial difficulties based on a longitudinal design. Our clinical experience teaches us that the psychosocial difficulties of persons with brain disorders fluctuate with the stage of the disease, not infrequently over short periods of time. It is common, for example, to observe changes in the levels of energy and drive, fluctuations in emotionality and in the extent to which the work performance is affected by the disease. An open question so far is the extent to which those short-term fluctuations at any stage of the disease process affect general health outcomes. This kind of information would be of considerable value for interventions and health systems planning.

The aim of this study is, therefore, to identify a set of psychosocial difficulties or disabilities using the ICF that are associated with short term changes in health outcomes across heterogeneous brain disorders.

## Methods

### Study design

As part of a three year European Commission funded FP6 coordination action involving nine European countries called ‘Measuring Health and Disability in Europe: supporting policy development’ (MHADIE http://www.mhadie.it), our study was a longitudinal observational investigation on a convenience sample of persons with the verified diagnosis of one of the following brain disorders: bipolar disorders (BD), depression, migraine, multiple sclerosis (MS), parkinson’s disease (PD), stroke and traumatic brain injury (TBI).

The disorders selected for this study are both neurological and psychiatric in nature and heterogeneous with respect to etiology, biochemical basis and signs and symptoms. In addition, all of these disorders either have a high global burden rating or are highly disruptive to a person´s life.

**Figure 1:**
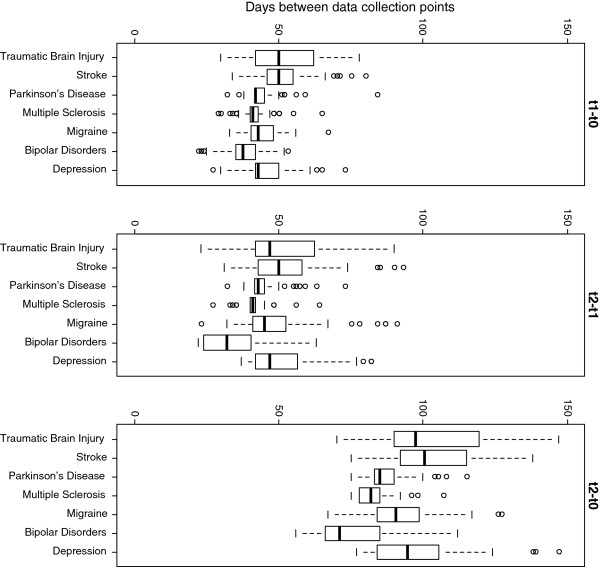
Boxplots showing the number of days between data collection time points.

The data for this study was collected in five study centers in four countries. One study center was in the Czech Republic, Italy, Slovenia and two in Spain. Data collection was performed at three time points: baseline (T0), approximately six weeks later (T1), and approximately twelve weeks later (T2). Since the time between time points was irregularly spaced, actual time in days from baseline is used as a time variable. Data on T1 was collected between 22 and 84 days after baseline with a mean of 44.17 and a median of 42 days, data on T2 between 55 and 147 days after baseline with a mean of 90.26 and a median of 88 days. Boxplots showing the number of days between data collection time points for each of the health conditions are presented in Figure 1.

The study protocol and the informed consent forms were approved by the responsible Ethics Committees at each center, namely the ethical committees of the Instituto Nazionale Neurologico “Carlo Besta” in Milan, Italy, the medical faculty of the Charles University in Prague, Czech Republic, the Institute for Rehabilitation in Ljubljana, Slovenia and the Hospital Universitario de La Princesa in Madrid, Spain. The inclusion criteria for patients were that the main diagnosis was one of the seven selected brain disorders; that the patient was at least 18 years old and had sufficient facility with the language of the country to understand all aspects of the study for purposes of consent; and that, finally, the patient agreed to participate and had signed the informed consent form.

### Measures

The socio-demographic data recorded was gender, age, years of formal education and current work status. Disease characteristics were specific for each health condition and the diagnosis was based on ICD-10 criteria. The severity of each health condition was determined by the following measures: Young Rating Scale of Mania Total Score for BD; Beck Depression Inventory II - BDI for depression; Migraine Disability Assessment Questionnaire (MIDAS) for migraine; Kurtzke Expanded Disability Status Scale (EDSS) for MS; Hoehn and Yahr Scale for PD; the Rankin Scale for stroke; and Functional Independence Measure (FIM) for TBI. The assignment of a patient to a severity grade (Mild, Moderate, Severe) was based on the distribution of the sample for the corresponding measure (Table 1).

**Table 1:** Measures used to determine the severity for each brain disorder, and the assignment to the severity scale according to the distribution of the sample

	***Measures***	***High Disease Severity***	***Moderate Disease Severity***	***Mild Disease Severity***
***Brain Disorders***				
*Bipolar Disorders*	Young Rating Scale of Mania Total Score	> 19 - Manic-Episode Group	≥ 7 and ≤ 19 - Subclinical and Hypomania Group	< 7 - Euthymia Group
*Depression*	Beck Depression Inventory II - BDI	> 28	≥ 14 and ≤ 28	< 14
*Migraine*	Migraine Disability Assessment Questionnaire (MIDAS)	> 20	≥ 6 and ≤ 20	< 6
*Multiple Sclerosis*	Kurtzke Expanded Disability Status Scale (EDSS)	> 5	≥ 3 and ≤ 5	< 3
*Parkinson's Disease*	Hoehn and Yahr Scale	= 3 or =4	= 2 or = 2.5	= 1 or = 1.5
*Stroke*	Rankin Scale	= 4 or =5	= 2 or = 3	= 0 or = 1
*Traumatic Brain Injury*	Functional Independence Measure (FIM)	>125	≥ 115 and ≤125	< 115

The data on disability was collected with the ICF checklist [16]. This checklist is a selection of 128 categories from each of the four components of the ICF – body functions (b), body structures (s), activities and participation (d), and environmental factors (e). It has been extensively used in several studies involving the application of the ICF. The following are examples of ICF categories from the different components: b134 Sleep functions, s410 Structure of cardiovascular system, d330 Speaking and e310 Immediate family. To facilitate the reading of the paper, we will not use the codes of the ICF categories but only their title from now on.

The severity of a problem for each *b, s* and *d* category was quantified by the ICF qualifier scale with response options 0 (NO problem), 1 (MILD), 2 (MODERATE), 3 (SEVERE), and 4 (COMPLETE problem). For the *e* component, the extent to which the category was a barrier was quantified on a comparable 0 to 4 scale. To denote an *e* category as a facilitator a positive sign (e.g. +2) was added. The option 8 was used when the available information was not sufficient to quantify the severity of the problem, and the option 9 was used when the category was not applicable to the patient.

For outcome measures, patients completed the Medical Outcome Study Short Form 36 (SF-36) [17] and the World Health Organization Disability Assessment Schedule (WHODAS II) [18,19]. Both of these were used as self-reported measures. The SF-36 is summarized by a Physical Component Summary measure (PCS, range 0–100, with higher values indicating better physical functioning) and a Mental Component Summary measure (MCS, range 0–100, with higher values indicating better mental functioning). In addition, the General Health (GH) Scale from the SF-36 was used as summary for the patient’s rating of his or her general health (range 0–100, with higher values indicating better general health).

The WHODAS II is a 36-item instrument developed to assess activity limitations and participation restrictions in six domains (understanding and communicating, getting around, self-care, getting along with people, life activities, and participation in society). Scores in the six domains as well as a global disability level score from 0 (best) to 100 (worst) are provided.

### Data collection procedures

Patient recruitment and data collection were performed by health professionals at each study center. The health professionals were trained for this in a structured three day workshop performed at Neurological Institute C. Besta Foundation IRCCS, Milan, Italy [20]. Consecutive patients in the five study centers mentioned above were recruited from September 2005 to May 2008 by the trained health professionals, who were psychologists, neurologists, psychiatrists and rehabilitation doctors.

The rating of the extent to which the patients had problems in the ICF categories or the extent to which environmental factors were barriers or facilitators was based on the information obtained from structured interviews held with each patient individually, as well as from clinical records. As advised by the WHO, for each patient only those ICF categories that are relevant were rated. After documentation of the demographic information and of the ICF categories contained in the ICF checklist, the patients were asked to fill in the self-reported questionnaires.

#### Data analysis

Descriptive statistics were used to characterize the study population, and to describe, at each time point of assessment, the four health outcomes, namely, the GH Scale, the PCS, and the MCS of the SF-36 and the WHODAS II global disability level score.

Boxplots were used to depict the change in within-person variation of health outcomes from T0 to T1, from T1 to T2 and from T0 to T2.

#### Data preparation

1) Response option 9 was set to 0 since when an ICF category is not applicable for a person, it can be assumed that it is not a problem.

2) Response option 8 was set to 1 since it indicated the presence of a problem but without specifying its extent.

3) The ICF categories with >50% missing values were excluded from the analyses.

4) The missing values of the remaining ICF categories were replaced five times by the Expectation-Maximization (EM) algorithm. The percentage of the missing data in each of these ICF categories is presented in Table 2.

5) The response options for all ICF categories were recoded 0–1, with 0 indicating no problem and 1 a problem at some level of severity. For e categories barrier was recoded −1, not relevant 0 and facilitator 1.

6) The five imputed datasets were separately prepared and the resulting datasets combined into a single dataset used for the next step of the analyses.

**Table 2:** The percentage of the missing data in each variable included in the model before multiple imputation*

**Labels**	**% missing T0**	**% missing T1**	**% missing T2**
Energy and drive functions	2.0	29.5	34.3
Sleep functions	0.4	26.5	32.0
Attention functions	0.3	26.5	32.0
Memory functions	0.4	26.5	32.1
Emotional functions	0.5	26.6	32.3
Sensation of pain	1.3	27.1	32.3
Weight maintenance functions	3.4	32.0	36.5
Watching	0.4	26.7	32.8
Listening	2.4	29.5	35.1
Solving problems	1.2	27.6	33.3
Communicating with - receiving - spoken messages	0.7	26.9	32.7
Speaking	2.1	28.8	34.1
Conversation	0.1	26.7	32.3
Lifting and carrying objects	1.1	28.3	34.3
Walking	0.9	26.9	32.3
Using transportation	2.4	31.3	36.2
Washing oneself	0.5	26.6	32.0
Caring for body parts	1.3	29.9	35.8
Dressing	0.7	27.1	32.9
Eating	0.7	26.9	32.4
Looking after one's health	2.2	28.4	34.4
Acquisition of goods and services	0.5	27.9	33.5
Preparing meals	1.3	29.8	35.6
Doing housework	0.8	29.2	34.1
Informal social relationships	1.5	27.9	32.5
Family relationships	1.2	30.0	34.9
Intimate relationships	1.3	29.1	34.4
Remunerative employment	1.6	27.4	33.5
Economic self-sufficiency	1.1	28.3	34.4
Community life	2.4	32.0	37.0
Recreation and leisure	1.3	27.4	33.1
Products or substances for personal consumption	0.5	34.1	39.4
Immediate family	1.3	27.8	34.0
Individual attitudes of immediate family members	2.2	30.6	36.1
Health services, systems and policies	1.5	31.1	37.8

* For each ICF category, at least one value of the three time points of assessment was available for each patient.

#### Multilevel model for change

Multilevel models for change were performed using the following steps to determine which ICF categories explain the variability and change over time of the four health outcomes in brain disorders (GH Scale, the PCS and the MCS of the SF-36 as well as the WHODAS II score).

1) An individual growth model (the level-1 component of the multilevel model) was fitted to describe the overall trend over time in our health outcomes. This method estimates the average trajectory as well as individual trajectories, thereby allowing the explicit examination of inter-individual differences in intra-individual change.

2) A level-2 submodel of the multilevel model that describes how the changes vary across patients was fitted introducing the time-invariant variables (age, gender and health condition) and the time-varying variable (disease severity) as predictors. They were kept in the model whether or not they had significant influence. The reference value for gender was male, the reference population for brain disorders was stroke, and the reference value for disease severity was the highest level of severity.

3) To identify which ICF category was the most predictive confounding variable or effect modifier of the health outcome, each ICF category was introduced (both itself and in interaction with time) in the model that resulted from step 2. Thus, as many models as ICF categories were created. The smaller the Akaike's Information Criterion (AIC) of the model the better predictor the corresponding ICF category. For the best predictor, the best model was selected based on the following criterion:

If a) both estimates for the ICF category itself and for the interaction with time were significant, or b) only the interaction with time was significant, then both terms were entered in the model. If c) only the estimate for the ICF category itself was significant, then only this term was entered in the model.

The resulting model was used as a starting point for the next step.

To identify in which sequence further ICF categories were introduced in the final model, step 3 was repeated for the remaining ICF categories based on the preceding model already containing the ICF categories selected in previous steps.

4) The selection procedure was stopped if the ICF category last entered into the model was not significant or if an ICF category already included into the model became non-significant.

Data analysis was performed with SAS version 9.2.

## Results

The demographic information of the study population as well as the descriptive statistics of the four health outcomes at each time point of assessment are presented in Table 3.

**Table 3:** Study population demographic information and descriptive statistics of the four health outcomes at each time point of assessment

**Patient characteristics**	**N = 741**			
Female patients; n (%)	498 (53.7%)			
Age: years; mean (sd)	50 (15.6)			
Yrs of formal education: yrs; mean (sd); median	13 (4.4); 13			
Current occupation				
Government employee; %	12.6			
Non-government employee; %	24.08			
Self-employed; %	7.9			
Employer; %	1			
Not working for pay; %	54.4			
	Health outcomes based on SF-36 and WHODAS II Score			
Time of assessment	GH Scale (N = 445)	PCS (N = 402)	MCS (N = 402)	WHODAS II Score (N = 415)
Time 0; mean (sd)	52.60 (21.70)	45.79 (10.35)	40.14 (14.27)	26.89 (19.72)
Time 1; mean (sd)	56.19 (21.33)	45.78 (10.29)	43.23 (12.02)	21.11 (17.13)
Time 2; mean (sd)	56.18 (21.17)	45.60 (10.52)	44.84 (11.43)	19.98 (16.69)

GH = General Health; PCS = Physical Component Summary; MCS = Mental Component Summary.

The data of 741 patients with brain disorders was collected. Depending on the amount of missing values in each health outcome, the corresponding sample sizes in the different models were different: 445 for GH Scale, 402 for MCS, 402 for PCS, and 415 for WHODAS II score. The distribution of persons per health outcome and condition is presented in Table 4.

**Table 4:** Distribution of persons per health outcome and condition

**Brain Disorder**	**GH Scale**	**MCS**	**PCS**	**WHODAS II Score**
Bipolar Disorders	73	73	73	74
Depression	63	62	62	64
Migraine	84	84	84	81
Multiple Sclerosis	36	35	35	37
Parkinson's Disease	60	60	60	44
Stroke	77	36	36	65
Traumatic Brain Injury	52	52	52	50
**Total**	**445**	**402**	**402**	**415**

**Figure 2:**
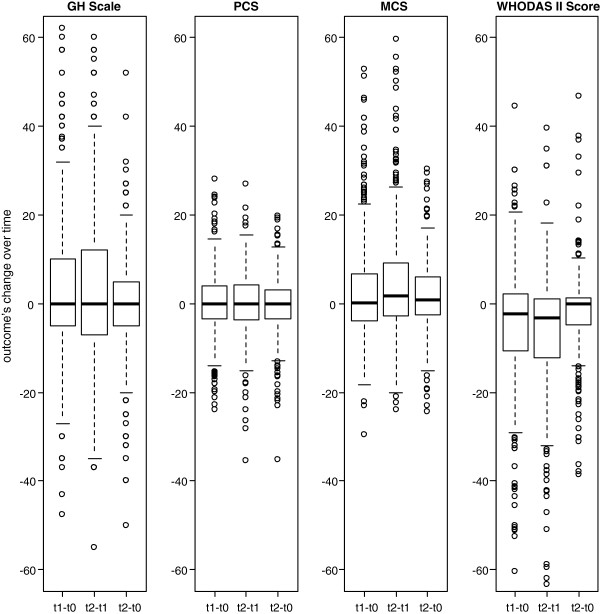
Boxplots showing extent of change in GH Scale, PCS, MCS and WHODAS II Score over time.

The boxplots presented in Figure 2 show how much change there is in the self-reported health outcomes over time.

One hundred and three ICF categories had >50% of missing values and were excluded from the analyses. The EM algorithm was used to replace missing values in the remaining 35 ICF categories which were used as potential predictors in the multilevel model for change: 7 ICF categories from the component *body function*s, 24 ICF categories from the component *activities and participation*, and 4 ICF categories from the component *environmental factors*.

Using the individual growth model, the development over time in the MCS and WHODAS II score can be described by a “linear change trajectory”, while in the GH Scale by a “quadratic change trajectory”.

Since time was not a significant predictor in the individual growth model for PCS, no multilevel model for change was calculated for this health outcome.

The three final multilevel models for change for GH Scale, MCS and WHODAS II score are presented in Table 5. For each of the independent variables included in the model the parameter estimate and its standard error (SE) are reported. Significant coefficients are marked in the table with * or **.

**Table 5:** Final multilevel models for change for each health outcome

	**GH Scale**		**MCS**		**WHODAS II Score**	
	**Estimate**	**SE**	**Estimate**	**SE**	**Estimate**	**SE**
Intercept	55.50**	2.58	32.20**	1.94	31.22**	1.73
Time in Days	0.06*	0.02	0.07**	0.01	−0.02*	0.01
Time in Days squared	−0.0006*	0.0002				
Gender	0.23	1.76	−1.55	0.85	−0.89	1.14
Age	−0.17*	0.06	−0.04	0.03	0.08*	0.04
Bipolar Disorders	8.92*	3.24	13.62**	1.71	−13.87**	2.07
Depression	3.43	3.31	5.34*	1.94	−3.64	2.11
Migraine	7.66*	3.11	12.82**	1.64	−16.63**	2.03
Multiple Sclerosis	−1.58	3.71	10.66**	1.95	−15.60**	2.40
Parkinson’s Disease	2.29	3.10	17.56**	1.63	−22.81**	2.04
Traumatic Brain Injury	18.36**	3.48	19.49**	1.89	−18.71**	2.26
Mild Disease Severity	6.96**	1.57	5.69**	0.87	−8.10**	1.05
Moderate Disease Severity	3.01*	1.33	2.96**	0.77	−3.57**	0.94
Energy and drive functions	−4.73**	1.10	−2.60**	0.64		
Sleep functions			−2.43**	0.59		
Emotional functions	−3.73*	1.18	−4.33**	0.69		
Sensation of pain			2.15*	0.70		
Solving problems			−3.48**	0.66	7.75**	1.02
Solving problems * Time in days					−0.03*	0.01
Conversation					4.59**	0.83
Lifting and carrying objects	−4.13*	1.17				
Walking	−4.54**	1.15			7.67**	0.79
Using transportation	−4.06*	1.58				
Washing oneself					8.28**	0.91
Acquisition of goods and services			−1.99*	0.68		
Informal social relationships	−3.95*	1.24			5.21**	0.89
Community life	−5.54**	1.22	−3.64**	0.68		
Recreation and leisure			−2.57**	0.63		
Products or substances for personal consumption			4.72**	1.06		
Products or substances for personal consumption * Time in days			−0.07**	0.01		
Health services, systems and policies	3.05*	1.01				

*P < 0.05, **P < 0.001.

Column ‘Estimate’ contains the mean of the regression coefficients and the column ‘SE’ the standard error of those estimates.

The estimates have to be interpreted with respect to the reference values, which were male for gender, stroke for the brain disorder, and ‘high’ for disease severity. For the ICF categories the reference value is not having a problem.

While positive estimates in the GH Scale and MCS indicate better health, they indicate lower health in the WHODAS II score.

The SE has to be interpreted in such a way that the smaller the SE, the more precise is the estimation of the regression coefficients.

The parameter estimate and the standard error (SE) for each of the independent variables are reported.

Age was a significant predictor for GH Scale and WHODAS II score, with higher age being related to worse health.

Compared to stroke, the reference health condition, the regression coefficients for all other brain disorders indicate significantly better health for MCS. For WHODAS II score, the regression coefficients for all other brain disorders, except depression, indicate significantly better health. Regarding the GH Scale, the regression coefficients for bipolar disorders, migraine and TBI indicate significantly better health than for stroke.

Four ICF categories of the component *body functions* were selected as significant predictors in relation to at least one health outcome, namely *Energy and drive functions* and *Emotional functions* for GH Scale and MCS, *Sleep functions* for MCS and *Sensation of pain* for MCS. All estimates but the one of *Sensation of pain* indicate that problems in these ICF categories are associated to lower health.

*Attention functions*, *Memory functions* and *Weight maintenance functions* were the body functions which were included in the analyses but not selected as significant predictors.

In the component *activities and participation*, 10 out of the original 24 ICF categories were selected as significant predictors for at least one of the three health outcomes. *Solving problems* was a significant predictor for MCS and WHODAS II score and also of the rate of change in WHODAS II score.

*Conversation* and *Washing oneself* were significant predictors for WHODAS II score, *Lifting and carrying objects* and *Using transportation* for GH Scale and *Acquisition of goods and services* and *Recreation and leisure* for MCS. *Walking* and *Informal social relationships* were significant predictors for GH Scale and WHODAS II score. Finally, *Community life* was a significant predictor for GH Scale and MCS.

For all selected ICF categories of the component *activities and participation* but the interaction term of *Solving problems* and time it applies that having a problem in them is related to poorer health.

The 14 ICF categories of the component *activities and participation* that were not significant predictors for any of the health outcomes were: *Watching*, *Listening*, *Communicating with -receiving - spoken messages*, *Speaking*, *Caring for body parts*, *Dressing*, *Eating*, *Looking after one's health*, *Acquisition of goods and services*, *Doing housework*, *Family relationships*, *Intimate relationships*, *Remunerative employment*, and *Economic self-sufficiency*.

In the component environmental factors, *Products or substances for personal consumption* and its rate of change were significant predictors for MCS and *Health services, systems and policies* was a significant predictor for GH Scale.

The two environmental factors that were not selected as significant predictors for any of the health outcomes were: *Immediate family* and *Individual attitudes of immediate family members*.

A comparison of multilevel models for change for each health outcome before introducing ICF categories in the model and for the final model is reported in Table 6. Variance estimates of random effects and goodness-of-fit statistics are reported for each model. For assessing goodness-of-fit, the models are compared to the corresponding individual growth model. For each parameter its mean and the mean of its standard errors (SE) over the five imputed datasets are reported. The deviance and the AIC reflect the fit of the models. Model fit always improves when adding the ICF categories.

**Table 6:** Comparison of multilevel models for change for each health outcome before introducing ICF categories in the model and for the final model

	**GH Scale**		**MCS**		**WHODAS II Score**	
	**Before introducing ICF categories**	**Final model**	**Before introducing ICF categories**	**Final model**	**Before introducing ICF categories**	**Final model**
Variance estimates of random effects: mean (SE)						
Intercept	267.81** (22.01)	211.96** (17.75)	52.27** (5.63)	35.94** (3.76)	127.63** (12.95)	72.65** (7.38)
Residual	130.02** (6.61)	115.79** (5.90)	57.41** (3.09)	42.24** (2.25)	78.08** (4.15)	55.42** (2.95**)**
Goodness-of-Fit: mean (SE)						
Deviance (−2 Log Likelihood)	10250.08 (3.98)	10110.68 (4.67)	8148.10 (2.60)	7798.55 (4.34)	8965.81 (3.27)	8496.80 (8.56)
Number of Parameters	16	24	15	25	15	21
Chi-square statistic(Degrees of freedom)	11.82** (10)	251.22** (18)	262.92** (10)	612.47** (20)	261.96** (10)	730.97** (16)
AIC	10282.08 (3.98)	10062.68 (4.67)	8118.10 (2.60)	7748.55 (4.34)	8935.81 (3.27)	8454.80 (8.56)

The deviance and Akaike’s Information Criterion (AIC). The smaller the deviance and the AIC the better the model fits the data.

*P < 0.05, **P < 0.001.

Variance estimates of random effects and goodness-of-fit statistics are reported for each model. For assessing goodness-of-fit the models are compared to the corresponding individual growth model. For each parameter its mean and the mean of its standard errors (SE) over the five imputed datasets are reported.

## Discussion

This study identified a set of psychosocial difficulties that are associated with short term changes in health outcomes in a group of heterogeneous brain disorders. These psychosocial difficulties are: sensation of pain, mental functions such as energy and drive functions, sleep and emotional functions, as well as a broad range of activities and participation domains, including solving problems, conversation, areas of mobility and self-care, relationships, community life and recreation and leisure. In clinical practice, these difficulties can be used as the basis for a description of the difficulties in people’s lives associated to these conditions and to make comparisons among them. In this context it is important to mention that all identified ICF categories (with the exception of vision) were included in the World Health Survey (WHS) used by the WHO in its 2000 report on health system performance assessment [21] and in the World Mental Health Surveys [22], in both cases to compare disability across conditions at the population level. The WHO set of domains (mobility, self-care, pain and discomfort, cognition, interpersonal activities, vision, sleep, energy, and affect) has not been further validated across populations. Even though the validation of the WHS set of domains was not an aim of this study, our results do show that these domains are relevant to a broad range of heterogeneous clinical samples with brain disorders.

The fluctuations in the health outcomes GH Scale, MCS and the WHODAS II score when measured over short periods of time were small. This means that to determine whether greater changes over time in the disorders take place, cohort studies that followed patients over much longer periods of time, most likely years, would be needed. Large sample studies of this sort involving patients with a number of brain disorders have not been published, as far as we are aware. There is, however, at least one coordination action at the European level currently being carried out to address the changes in psychosocial difficulties of brain disorders over time (PARADISE http://www.paradiseproject.eu). PARADISE is currently testing an innovative approach to collecting clinical data on the psychosocial difficulties that people experience when they suffer from brain disorders, the determinants of the occurrence of those difficulties and the determinants of their change over time. The results of this project will be available mid-2013. In addition, the results show that it will be important to analyze whether both outcomes related to mental health and physical health vary over time and whether different psychosocial domains predict these changes.

All the models improved after introducing the ICF categories, and some of these results need special comment:

By introducing eight ICF categories – energy and drive functions, emotional functions, lifting and carrying objects, walking, using transportation, informal social relationships, community life and health services, systems and policies – to time, gender, age, health condition and disease severity, it is possible to explain the variation among patients in self-reported general health. This suggests that both bodily domains (energy and drive, emotional functions, carrying objects, walking and using transportation) and the more social domains (informal social relationships and community life) need to be considered in order to adequately describe the experience of general health for persons with brain disorders, and thereby to gain the advantages for intervention planning and other applications of a more extensive understanding of this experience.

Similarly, model fit of self-report mental health was shown to be improved after introducing a broad range of variables that included sleep functions, solving problems, conversation, acquisition of goods and services and drugs. These results are especially interesting since none of the psychosocial domains mentioned are included in the SF-36 [23], which underscores the importance of information about social domains, as well as mental domains, for the overall description of mental health in brain disorders.

The psychosocial difficulties that contribute to the improvement of the model fit of the WHODAS II summary score are difficulties covered by questions in this questionnaire, so it should not come as a surprise that they were selected in the regression model. It is important to mention that solving problems helps to explain the variability over time of the WHODAS II summary score. Solving problems is a domain of activity and participation that is not consistently taken into account in studies of people experiencing brain disorders. Our investigation provides some evidence that this domain is an important psychosocial difficulty relevant to a comprehensive description of the experience of living with a brain disorder.

The relationship between the disability measured with the WHODAS II and with the SF-36 in brain disorders has already been evaluated in two papers on depression and migraine [24,25]. Results from these studies account for mild to moderate correlations between these questionnaires, thus confirming that both measure difficulties related to different psychosocial problems, and that measures of psychosocial difficulties should be employed in clinical and public health research using a cross-cutting perspective, and not only a disease-specific one.

Finally, this study shows that it is useful to use the ICF as a source of independent variables for longitudinal studies and as a basis for defining psychosocial difficulties. Using ICF made it possible for us to analyze together the data derived from different brain disorders. Psychosocial difficulties were conceptualized in terms of ICF as disabilities arising from the interaction of the brain disorder and personal and environmental factors. They represent impairments, activity limitations and participation restrictions. Impairments and activity limitations are decrements of functioning capacity, and so are elements of a person’s health state. Participation restrictions go beyond the health state to include restrictions in a person’s performance of actions, tasks and behaviors in his or her actual environment [26].

We have shown that psychosocial difficulties must not be defined solely in terms of the health conditions that produce decreases in functioning in various domains, but also in terms of the physical, social and attitudinal environment, which, when taken into account, contributes to explain the impact of brain disorders on people’s lives. The social and attitudinal environment, especially in the case of brain disorders, includes barriers such as fear, misunderstanding, stigma and discrimination [27-30], as well as the absence of social policies to accommodate people with brain disorders in the workplace and elsewhere in society [31,32].

Against our expectations, in this study three environmental factors - Immediate family, Individual attitudes of immediate family members and Health services, systems and policies - were not selected in the corresponding models as significant independent variables. There are potentially many reasons for this, including of course limitations of our study design, such as having to collapse the response options of the ICF qualifiers from 0 to 4 to the dichotomous, 0 and 1. Doing so resulted in a loss of variability, and consequently sensitivity to change. The collapsing was performed because model complexity would have greatly increased if the ICF categories had been used as categorical variables with 5 and 9 response options, respectively, and for each response option, interaction with time would have to have been separately considered. A second limitation of the study that should be mentioned is the irregularity with which the time points of assessment are spaced. This irregularity was due to practical considerations, such as the time in which the health professionals could access the patients’ clinical records and performed the interviews. Nevertheless, the irregular time points of assessment across patients do not put into question the validity of the results, since time in days has been included as a metric variable in the multilevel models for change. Finally, 103 ICF categories had >50% of missing values and were excluded from the analyses. As mentioned before, we followed the recommendations of WHO and only those ICF categories that are relevant to the patient are documented. Thus, we assume that the ICF categories excluded from the analyses refer to issues that are not relevant for the large majority of the patients and therefore the validity of the results is not compromised. However, we cannot deny that the decisions of the health professionals regarding the relevance for the patients of areas addressed by ICF categories is fallible, which might have influenced the validity of the data at the end. For future studies, we recommend that the same ICF categories always be evaluated for all the patients included in the study.

## Conclusion

Our findings are of primary interest to researchers and clinicians as they summarize a comprehensive list of relevant psychosocial difficulties that explain the variability in health outcomes. Our study also indicates that there is an explanatory role for fundamental human relationships in accounting for changes over time. In addition to difficulties that are frequently part of diagnostic criteria, such as emotional and energy and drive functions, there are other fundamental difficulties that a wide range of brain disorders share. This study is an early attempt to identify the influence of these difficulties over time using the comprehensive framework of the ICF. Further longitudinal studies are obviously needed to map out both the patterns and the variations in the experience of living with a brain disorder.

## Competing interests

The authors declare that they have no competing interests.

## Authors’ contributions

AC planned the study and provided the first draft of the manuscript, CB and CO carried out the statistical analyses, JLAM, AR, ML and EV took care of the data collection and contributed to the planning of the study, JB and SC supervised the study and contributed to the several revisions of the manuscript. All authors read and approved the final manuscript.

## Pre-publication history

The pre-publication history for this paper can be accessed here:

http://www.biomedcentral.com/1471-244X/13/78/prepub
